# Antifungal Activity and Action Mechanism of Ginger Oleoresin Against *Pestalotiopsis microspora* Isolated From Chinese Olive Fruits

**DOI:** 10.3389/fmicb.2018.02583

**Published:** 2018-10-30

**Authors:** Tuanwei Chen, Ju Lu, Binbin Kang, Mengshi Lin, Lijie Ding, Lingyan Zhang, Guoying Chen, Shaojun Chen, Hetong Lin

**Affiliations:** ^1^College of Food Science, Fujian Agriculture and Forestry University, Fuzhou, China; ^2^Fujian Bio-Engineering Professional Technology Institute, Fuzhou, China; ^3^Food Science Program, Division of Food System & Bioengineering, University of Missouri, Columbia, MO, United States; ^4^U.S. Department of Agriculture, Agricultural Research Service, Eastern Regional Research Center, Wyndmoor, PA, United States

**Keywords:** Chinese olive (*Canarium album* Lour.), fruit, pathogenic fungi, *Pestalotiopsis microspora*, ginger oleoresin, antifungal activity, action mechanism

## Abstract

*Pestalotiopsis microspora* (*P. microspora*) is one of dominant pathogenic fungi causing rotten disease in harvested Chinese olive (*Canarium album* Lour.) fruits. The purposes of this study were to evaluate the antifungal activities of ginger oleoresin (GO) against *P. microspora* and to illuminate the underlying action mechanisms. The *in vitro* assays indicate that GO exhibited strong antifungal activity against mycelial growth of *P. microspore*, and with 50%-inhibition concentration (*EC*_50_) and 90%-inhibition concentration (*EC*_90_) at 2.04 μL GO and 8.87 μL GO per mL propylene glycol, respectively, while the minimal inhibitory concentration (MIC) and minimal fungicidal concentration were at 10 μL GO and 30 μL GO per mL propylene glycol, respectively. Spore germination of *P. microspora* was inhibited by GO in a dose-dependent manner, and with 100% inhibition rate at the concentration of 8 μL GO per mL propylene glycol. Compared to the control, the cellular membrane permeability of *P. microspora* increased due to severe leakage of intercellular electrolytes, soluble proteins, and total sugars with the treatments (*EC*_50_, *EC*_90_) by GO during incubation. In addition, analysis of fatty acid contents and compositions in cellular membrane by GC-MS indicated that GO could significantly promote the degradation or peroxidation of unsaturated fatty acids in *P. microspore*, resulting in the enhancement of membrane fluidity. Moreover, observations of microstructure further showed the damage to plasma membrane and morphology of *P. microspora* caused by GO, which resulted in distortion, sunken and shriveled spores and mycelia of the pathogen. Furthermore, *in vivo* assay confirmed that over 3 MIC GO treatments remarkably suppressed disease development in *P. microspore* inoculated-Chinese olive fruit. These results demonstrate that owing to its strong antifungal activity, GO can be used as a promising antifungal agent to inhibit the growth of pathogenic fungi in Chinese olives.

## Introduction

Chinese olive (*Canarium album* (Lour.) Raeusch), a widely consumed subtropical fruit, is endemic to southeast China. It has a fusiform drupe and is in yellowish green similar to Mediterranean olive (*Olea europaea* L.), but has a relatively low oil content (Zhan et al., 2015). Matured Chinese olive fruits are usually consumed fresh or processed by the food industry to beverages, candy, and other products that conserve high nutritional values. They possess great pharmacological functions such as detoxification and inhibition against bacteria, virus, inflammation, and oxidation (He et al., 2008; Kuo et al., 2015; Chang et al., 2017; Lin et al., 2017). However, unfortunately, putrefaction can develop due to pathogenic infections in the harvested fresh fruits of Chinese olive, which may result in considerable quality losses and a shorter shelf life. A long list of pathogens has been reported causing postharvest infectious diseases of Chinese olive fruits, including *Pestalotiopsis microspore*, *Fusarium oxysporum* Schlecht., *Monochaetia karstenii* (Sacc & Syd.) Sutton, *Pestalotiopsis eriobotrya folia* (Guba) Chen et Chao, *Glomerella cingulata* (Stonem.) Spauld. Et Schrenk, *Colletotrichum gloeosporioides* Penz., *Botryodiplodia theobromae* Pat., *Penicillium* sp., and *Phytophthora palmivora* (Butl.) Butler (Chen et al., 2015, 2016a,b). Our previous studies demonstrated that *P. microspora* is a dominant pathogenic fungus that can make fruits rot (Chen et al., 2016b).

To date, traditional chemical fungicides such as prochloraz, thiophanate methyl, and carbendazol have been extensively used to combat infectious diseases in postharvest Chinese olive fruits. However, pesticide residues in fruits can lead to harmful effects on human health and the development of fungicide resistance in pathogens. Hence, there remains a need to develop safer, more effective and eco-friendly alternative fungicides that cause minimal damage to the environment and human health.

The use of botanical fungicides was considered a viable and better alternative approach for the control of pathogenic fungi because effective control of a variety of rot pathogens in diverse foods have been reported (Nanasombat and Wimuttigosol, 2011; Jayasena and Jo, 2013; Bag and Chattopadhyay, 2015; Chaemsanit et al., 2018). For example, ginger oleoresin (GO), a complex mixture extracted from ginger (*Zingiber officinale* Roscoe), is rich in gingerols and shogaols. Some previous studies showed that GO had good capability of inhibiting the growth of certain types of fungi, such as *Aspergillus* species, *Fusarium moniliforme*, *Fusarium verticillioides*, *Rhizoctonia solani*, *Cryptococcus Neoformans*, *Candida albicans*, and *Penicillium spp.* (Singh et al., 2008; Yamamoto-Ribeiro et al., 2013; Bellik, 2014; Ashraf et al., 2017; Varakumar et al., 2017). However, to the best of our knowledge, little information is available on the antifungal activity and the mode of action of GO against *P. microspora* in Chinese olives. Thus, this study aimed to verify a hypothesis that GO is a potent antifungal agent against *P. microspore* in Chinese olives.

The main goal of this study was to investigate the effects of GO *in vitro* on the growth of *P. microspora* in Chinese olives. In addition, the changes of structures and components of cell membrane were also evaluated to elucidate the possible antifungal mechanism of GO. Moreover, antifungal effects of the GO treatment on the *in vivo* disease development of *P. microspore* in Chinese olive fruits were also evaluated.

## Materials and Methods

### Preparation of *P. microspora* Spore Suspension

*Pestalotiopsis microspora* (GL-3) was isolated from Chinese olive (*Canarium album* Lour. cv. Changying) fruit via tissue isolation and identified using the methods of morphology, molecular biology, and phylogenetic analysis as described by Chen et al. (2016a). *P. microspora* (GL-3) was preserved at Institute of Postharvest Technology of Agricultural Products, College of Food Science, Fujian Agriculture and Forestry University, Fuzhou, China.

The preparation of *P. microspora* spore suspension was based on the method of Chen et al. (2016b). Briefly, *P. microspora* was inoculated in autoclaved potato dextrose agar (PDA) medium for activation, and then transferred to oat bran medium (OB, contains 60 g oat flour, 60 g rice bran flour, 20 g sugar, and 20 g agar per liter) for inoculation for 7 d at 28°C. The plates were then washed with sterile 0.9% of NaCl and the solutions were transferred into sterile conical bottles and gently shaken to release spores. Finally, the spore suspensions were filtered through multilayers of sterile gauze to remove mycelial fragments, and adjusted to 1 × 10^6^ spores mL^−1^ with the aid of a hemocytometer.

### Determination of Mycelial Growth Inhibition by GO

The measurement of the mycelial growth of *P. microspora* was conducted by two perpendicular directions method (Zhang et al., 2012). GO, which contains 25% (*m*/*v*) gingerols, was purchased from Yanyi Bio., Co., Ltd., Shanghai, China. Different concentrations of GO with 2, 4, 6, 8, 10, and 12 μL per mL propylene glycol were prepared, and added to autoclaved liquid PDA mediums, respectively, then cooled to obtain solid PDA medium with different concentrations of GO. PDA mediums containing 0 μL GO per mL propylene glycol served as the controls. A mycelial colony (5 mm in diameter) was cut from the edge of 5 d-old *P. microspora* colony and placed upside down on the center of the plate with fungi in contact with the growth medium. Cultures were incubated at 28°C for 5 d prior to measurement of the mycelial growth diameter (mm) of *P. microspora* in two perpendicular directions. The inhibitory rate of mycelial growth was calculated with the following formula:

$${\mathrm{IR}}_{\text{mg}}=\frac{\left(d_{\text{c}}-d_{\text{t}}\right)}{d_{\text{c}}-5}\times 100\%$$

where *IR*_mg_ was the inhibitory rate of mycelial growth, %;

*d*_c_ and *d*_t_ were average diameter (mm) of mycelial colonies of the control and the GO treatment, respectively;

5 was the diameter (mm) of original mycelial colony.

In addition, the effective concentration for a 50% reduction (*EC*_50_) and 90% reduction (*EC*_90_) of mycelial growth was calculated according to the growth curves of the relationship between the GO concentration (μL per mL propylene glycol) and *IR*_mg_ (%).

### Determination of Spore Germination Inhibition Activity

Spore germination of *P. microspora* was detected with minor modifications as described by Pane et al. (2016). A volume of 20 μL of spore suspension containing 1 × 10^6^ spores mL^−1^ was incorporated into PDA mediums with 100 μL various concentrations of GO at 0, 2, 4, 6, 8, 10, and 12 μL per mL propylene glycol, respectively, and then cultured at 28°C for 7 h. The spore germination was observed by microscopy. The germination was determined when the length of a germ tube exceeded half of the small-end diameter of the spore, and at least 200 spores were examined in each visual field before determination. Spore germination rate was expressed as percentage of the germinated spores to the total calculated spores, and the inhibitory rate of spore germination was calculated by the following formula:

$${\mathrm{IR}}_{\text{sg}}=1-\frac{{\mathrm{IR}}_{\text{t}}}{{\mathrm{IR}}_{\text{c}}}\times 100\%,$$

where *IR*_sg_ was the inhibitory rate of spore germination, %;

*IR*_t_ and *IR*_c_ were represented as the inhibitory rate of spore germination at control and at GO treatment, respectively.

Each replicate consisted of three observations, and three replicates were performed for each treatment.

### Determination of the MIC and MFC

The minimal inhibitory concentration (MIC) and minimal fungicidal concentration (MFC) for *P. microspora* were determined by broth dilution method (Shukla et al., 2009). 20 μL spore suspension containing 1 × 10^6^ spores mL^−1^ was incorporated into PDA media with different concentrations of GO at 5, 10, 15, 20, 25, 30, and 35 μL per mL propylene glycol, respectively, and then cultured at 28°C for 7 d. MIC is the lowest concentration which did not support visible fungus. Mycelia from the plates showing no growth were sub-cultured on treatment-free PDA plates to determine if the inhibition was reversible. The lowest concentration at which no growth occurred was defined as MFC.

### Observation of Morphological Structures of Mycelia

The mycelial sample preparation for mycelia observation was based on a modified method of Tian et al. (2012). An aliquot of 1 mL spore suspension (1 × 10^6^ spores mL^−1^) was added to autoclave PDA medium containing GO at 0, *EC*_50_ and *EC*_90_. After 7 d incubation at 28°C, 10 mL fungal suspension was centrifuged at 10 000 r min^−1^ for 15 min at 4°C, the supernatant was discarded, and the precipitate was washed three times with sterile distilled water for microscopic observation.

The microstructural images of the as-prepared mycelia samples were observed using scanning electron microscopy (SEM) equipped with a JSM-6380LA microscope (JEOL, Japan) at an accelerating voltage of 15 KV. The mycelial samples were firstly fixed with 3–4% (*v*/*v*) glutaraldehyde at room temperature for 4–6 h and then washed five times with 100 m mol L^−1^ phosphate buffer (PBS, pH 7.0), post-fixed with 1% osmium tetroxide for 1.5 h. After washing with the same buffer twice, the specimens were dehydrated in a graded ethanol series (30, 50, 70, 80, 90, and 100%) for three times for 15 min in each series. Finally, the samples were dried in vacuum dryer (DZF6020, JingHong, Shanghai, China), then gold-coated and examined by SEM.

### Measurement of Cellular Leakage

The leakage of electrolytes of *P. microspora* was measured according to the method of Tian et al. (2015) with minor modifications. A volume of 1 mL spore suspension (1 × 10^6^ spores mL^−1^) was added into 100 mL autoclave potato dextrose broth (PDB) medium, follow by incubation at 28°C for 3 d, then the fungal cells were centrifuged at 10 000 r min^−1^ for 15 min at 4°C to obtain the supernatant. The extracellular conductivities (μS cm^−1^) of *P. microspora* cells supernatant were determined continuously using a DDS-307 electric conductivity meter (Jingke Scientific Instrument, Shanghai, China) after treatments with different GO concentration at 0, *EC*_50_ and *EC*_90_ for 0, 60, 120, 180, 240, and 300 min. Finally, the relative conductivity was used to reflect the cellular leakage of pathogen, which was calculated and expressed as the percentage of conductivity in treatment with GO as the control.

The leakage of the intracellular soluble proteins, total sugars and nuclein from mycelium of *P. microspora* was assayed according to the method of Li et al. (2018). A volume of 1 mL spore suspension containing 1 × 10^6^ spores mL^−1^ was cultured on 100 mL PDB medium for 5 d at 28°C and mycelium were harvested. The mycelium was collected and lyophilized (FDU-1200, EYELA, Tokyo, Japan) after being washed three time with sterile distilled water. Subsequently, 1.0 mg lyophilized mycelium were re-suspended in 0.1 mol L^−1^ PBS (pH 7.0) containing the addition of various GO concentration at 0, *EC*_50_ and *EC*_90_ and incubated at 28°C. Then, the fungal suspension was centrifuged at 12 000 r min^−1^ for 5 min after 2, 4, and 6 h incubation and the supernatant was collected for determination of the leakage of intracellular soluble proteins, total sugars, and nuclein. The total sugars content of mycelia of *P. microspora* was determined by anthrone-sulfuric acid method (Moshayedi et al., 2013) using glucose as the standard. Soluble protein content was determined according to the Bradford assay (Bradford, 1976) with bovine serum albumin as the standard. The leakage from cellular membrane was measured according to the absorbance at 260 nm (OD_260__nm_) by ultraviolet visible adsorption spectrometry (UV-1750, Shimadzu, Japan) using fungal suspension with only PBS as the control.

### Assay of Fatty Acid Composition in Cell Membrane

The change of fatty acid contents and compositions in cell membrane of *P. microspora* was analyzed by GC-MS (Tridion-9, Torion, United States). Preparation of fatty-acid methyl ester in cell membrane was performed according to Hazzit et al. (2006) with minor modifications. Aliquots of 20–30 mg mycelia were weighed into a 5 mL centrifuge tube, 1.0 mL of NaOH-MetOH, and 2.0 mL of HCl-methanol were added and kept in boiling water bath for 30 min, and then cooled to room temperature using ice bath. Next, the mixture was extracted with 1.25 mL hexane/ether (2:1, *v*/*v*) and allowed to stand at room temperature for 15 min for stratification. The organic phase was transferred to another tube and 3.0 mL of NaOH and few drops of saturated NaCl solution was added. The tubes were sealed and shaken back and forth for 10 min. Finally, 1 mL of organic phase was pipetted for GC-MS quantification of fatty acid. The indexes of unsaturated fatty acid were calculated and expressed as the percentage of contents of unsaturated fatty acid to contents of saturated fatty acid.

### Antifungal Activity *in vivo*

Healthy and uniform maturity “Changying” Chinese olives were obtained from an olive orchard in Fuzhou, Fujian, China. Antifungal experiment *in vivo* was conducted by injury inoculation with some minor modifications (Li et al., 2018). Firstly, fruits were surface-sterilized using 2% sodium hypochlorite solution (Sinopharm, Beijing, China) for 2 min and rinsed twice with sterilized distilled water, then air-dried. Next, the fruits were artificially injured using a sterilized hole punch to make a 5 mm × 3 mm (diameter × depth) wound on the surface, in which 15 μL freshly prepared spore suspension (1 × 10^6^ spores mL^−1^) was inoculated, follow by addition of 20 μL of GO with the concentration of 1, 2, 3, and 4 MIC. Finally, the treated fruits were incubated at 28°C and 95% relative humidity (RH) for 6 d. Fruit not treated with GO was used as the control. The lesion diameter (in mm) of fruit was measured using a vernier caliper every another day.

### Statistical Analysis

All experiments were repeated three time and data were acquired. The values in figures were expressed as the means and standard errors. Analysis of variance (ANOVA) was used to analyze the data using the software (SPSS version 17.0). Student’s *t*-test was used to compare the mean values of the data set. A *p*-value ≤ 0.05 or 0.01 was considered statistically significant.

## Results and Discussion

### Effect of GO on Inhibition Activity of *P. microspora* Mycelial Growth

**FIGURE 1:**
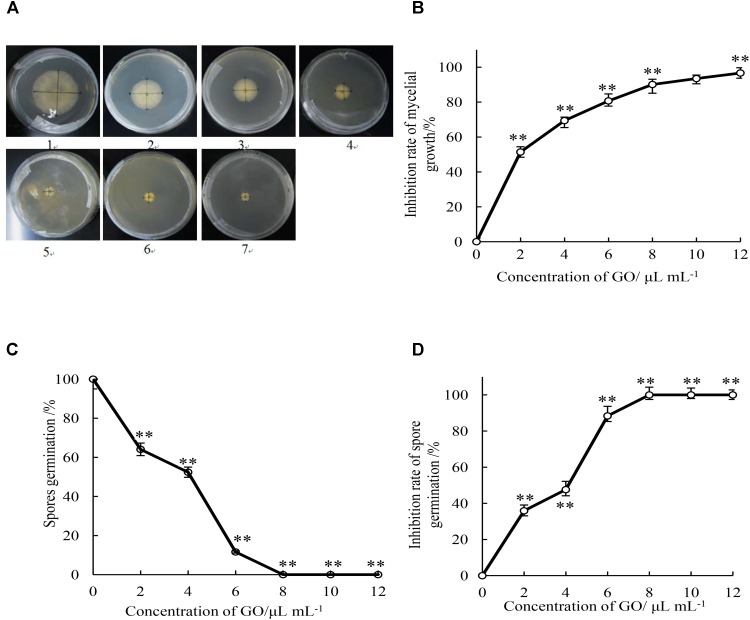
Effects of different concentrations of GO on colony diameter (mm) **(A)**, inhibition rate of mycelial growth **(B)**, spore germination rate **(C)**, and inhibition rate of spore germination **(D)** of *P. microspora.* Each value is the mean for three replicates. The vertical bar indicates the standard error. The asterisks indicate significant difference between control and GO treated fruit (^∗∗^*P* < 0.01). Plate of 1 in Figure 1A represented the treatment of propylene glycol was for control. Plates of 2 to 7 represented the treatments of GO with concentration of 2, 4, 6, 8, 10, and 12 μL per mL propylene glycol, respectively.

As shown in Figure 1A, the increase of the colony diameter of mycelial growth was comparatively slower in all treatments with GO than in the control medium during 5 d incubation period. Mycelial growth of *P. microspora* was significantly (*p* < 0.05) inhibited by GO in a concentration-dependent manner, the higher the concentration, the higher the inhibition rate (Figure 1B). Furthermore, the *EC*_50_ (2.04 μL mL^−1^) and *EC*_90_ (8.87 μL mL^−1^) of mycelial growth by the GO treatment was calculated according to the regression analysis (*y* = 2.0093x + 4.3759, *r* = 0.986), which indicated that *P. microspora* was inhibited effectively at low concentration.

### Effect of GO on Inhibition Activity of *P. microspora* Spore Germination

The spore germination rate of GO treatments with the concentration of 0, 2, 4, 6, 8, 10, and 12 μL per mL propylene glycol were determined after 7 h incubation (Figure 1C). Spore germination of *P. microspora* was significantly (*p* < 0.01) inhibited by various concentration of GO treatments. The spore germination rate reached 100% in the control after incubation for 7 h, it was inhibited by 35.9, 47.6, and 88.4% by GO concentration of 2, 4, and 6 μL per mL propylene glycol. Complete inhibition (100%) was achieved at GO concentrations beyond 8 μL per mL propylene glycol (Figure 1D).

### Determination of MIC and MFC

Based on the observation of mycelial growth on the PDA medium with GO treatments at 0, 5, 10, 15, 20, 25, 30, and 35 μL per mL propylene glycol during 7 d incubation period, the MIC and MFC values of GO treatment against mycelial growth of *P. microspora* were measured to be 10 and 30 μL per mL propylene glycol, respectively (Table 1).

**Table 1:** Results of minimal inhibitory concentration (MIC) and minimal fungicidal concentration (MFC) of GO *for P. microspora*.

Concentration of GO/μL mL^−1^	Mycelial growth for 2 days	Mycelial growth for 7 days	MIC/μL mL^−1^	MFC/μL mL^−1^
0	+	+	10	30
5	+	+		
10	−	+		
15	−	+		
20	−	+		
25	−	+		
30	−	−		
35	−	−		

*“ + ”,: presence of mycellia growth; “−”, absence of mycelia growth.*

### Effect of GO on Mycelial Morphology of *P. microspora*

**FIGURE 2:**
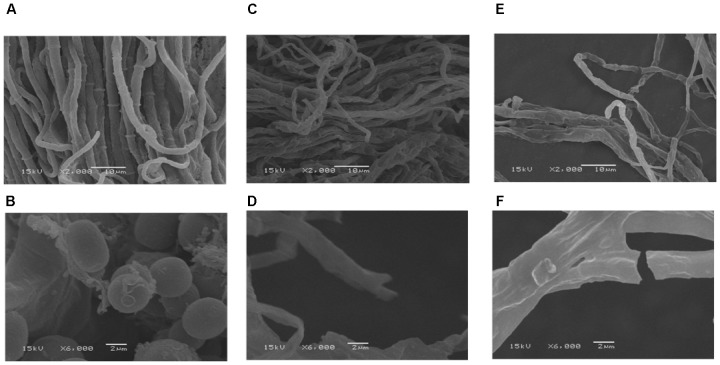
Scanning electron microscopy images with the bars at 2 μm and 10 μm of mycelia of *P. microspora* exposure to 0 **(A,B)**, *EC*_50_ **(C,D)** and *EC*_90_ **(E,F)** of GO.

Morphological observations by SEM exhibited that the control sample of *P. microspora* had smooth, uniform and vigorous mycelia (Figure 2A), with distinctive intercellular septa and broom-like structures with beaded conidium at the top (Figure 2B). However, the mycelia appeared evidently disordered, rough and sunken after exposure to 2.04 μL mL^−1^ (*EC*_50_) GO (Figure 2C); meanwhile, the intercellular septa disappeared and no conidium were observed in the structure (Figure 2D). Furthermore, the mycelia of *P. microspora* treated with 8.87 μL per mL propylene glycol (*EC*_90_) were greatly distorted, intertwined and crimpled (Figure 2E), which caused irregular constriction even disruption (Figure 2F). Therefore, the evidence from SEM observation indicated that the treatments with GO caused distortion, sunken and serious damage to the morphology of the mycelial and effectively inhibited its growth due to the serious destruction of integrity of mycelial structure that interfered physiological metabolism.

### Effect of GO on Membrane Permeability of *P. microspora*

**FIGURE 3:**
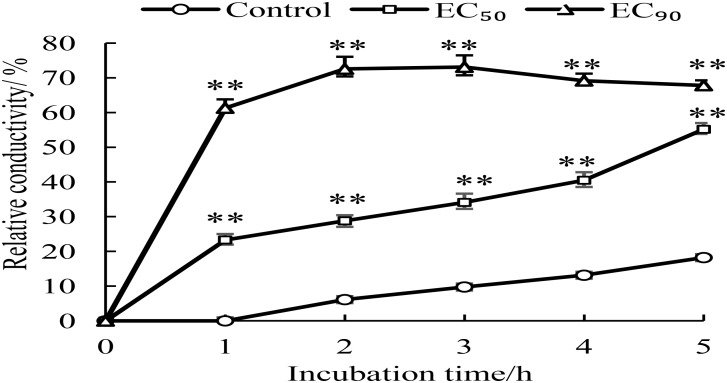
Effects of different concentrations of GO on extracellular conductivity of *P. microspora*. Each value is the mean for three replicates. The vertical bar indicates the standard error. The asterisks indicate significant difference between control and GO treated fruit (^∗∗^*P* < 0.01).

Changes of membrane permeability were considered as an indicator of damage of cell membrane structure (Li et al., 2018). The membrane permeability increased when the normal cells were destroyed, which caused variation of conductivity due to imbalance of intracellular and extracellular electrolytes. In general, the more serious the cell membrane damage, the higher the conductivity. So, the relative conductivities of mycelia treated for 1–5 h by GO were detected. As shown in Figure 3, the relative conductivity slowly increased overtime (0–5 h) in the control, which could be attributed to the autolysis of normal cell. However, the relative conductivities of the mycelia treated with GO exhibited obvious increase comparing to the control, especially, which reached highest value after 2 h and maintained at higher level by GO treatment with *EC*_90_ than that of *EC*_50_. In short, GO treatment accelerated the leakage of electrolytes in *P. microspore*, resulting in the great increase of membrane permeability.

### Effect of GO on Cellular Leakage of *P. microspora*

**FIGURE 4:**
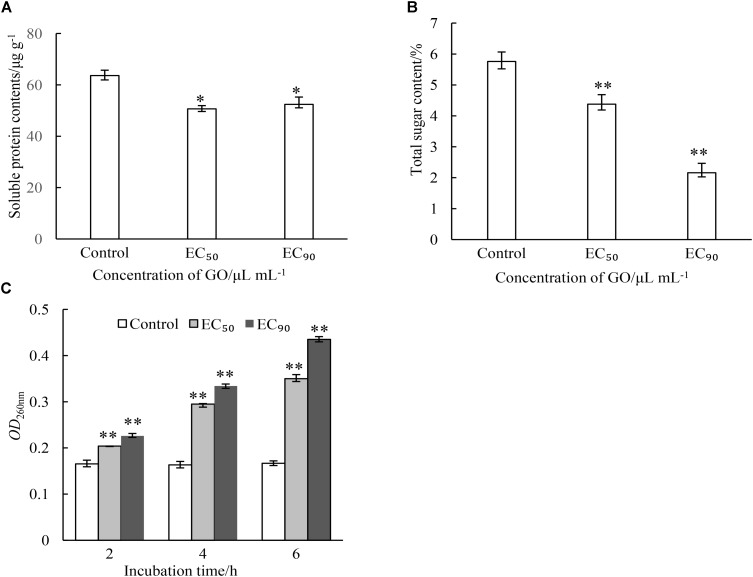
Effects of different concentrations of GO on leakage of proteins **(A)**, leakage of total sugars **(B)** and leakage of nuclein **(C)** of *P. microspora*. Each value is the mean for three replicates. The vertical bar indicates the standard error. The asterisks indicate significant difference between control and GO treated fruit (^∗∗^*P* < 0.01, ^∗^*P* < 0.05).

Intracellular substances are essential material bases for the growth and reproduction of microorganisms. This study evaluated the effects of GO on the contents of cellular substances including protein, total sugar and nuclein in mycelia of *P. microspora*. As demonstrated in Figure 4, GO treatments caused different degrees of leakage of the substances *via* cell membrane of *P. microspora* in the control. The content of protein in mycelia was not substantially affected by the concentration of GO, but significantly different (*p* < 0.05) from the control (Figure 4A). The contents of total sugar in mycelia decreased dramatically (*p* < 0.01) from 5.76 μg g^−1^ without GO to 4.38% and 2.16% with GO at *EC*_50_ and *EC*_90_, respectively (Figure 4B). Simultaneously, the longer the treating time, the greater the release of nuclein from mycelia, the absorbance of mycelial supernatant (*OD*_260nm_) increased dramatically to a value 2.45-fold, which is higher than that of the control after 5 h inoculation (Figure 4C). These results aligned with the morphological observations in this study.

### Effect of GO on Fatty Acid Composition of Membrane Lipids in *P. microspora*

**FIGURE 5:**
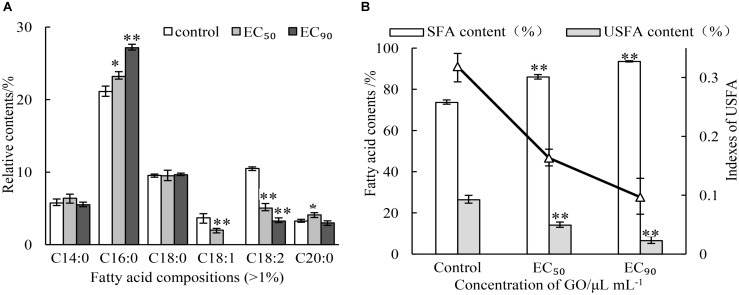
Effects of different concentrations of GO on fatty acid compositions **(A)** and indexes of unsaturated fatty acid **(B)**. Each value is the mean for three replicates. The vertical bar indicates the standard error. The asterisks indicate significant difference between control and GO treated fruit (^∗∗^*P* < 0.01, ^∗^*P* < 0.05).

As the main components of membrane, the contents and compositions of fatty acids affect the stability of membrane. The impacts of GO on fatty acid composition of membrane lipids were analyzed by GC-MS. The results were presented in Figure 5A. Six main kinds of fatty acids were found, including palmitic acid, linoleic acid, octadecanoic acid, tetradecanoic acid, oleic acid, eicosanoic acid in the normal cell of *P. microspora* after 3 d of incubation (control), where saturated fatty acids and unsaturated fatty acid were account for 73.63 and 26.37%, respectively. Nevertheless, the saturated fatty acids increased by treating with GO, but the opposite was true for unsaturated fatty acids. In particular, the oleic acid was not detected after treatment with GO at *EC*_90_. The changes of fatty acid composition (Figure 5B) also caused sharp decline of indexes of unsaturated fatty acid (IUFA) from 0.3186 (control) to 0.1632 (*EC*_50_), and 0.0697 (*EC*_90_). These findings indicate that GO could significantly promote the degradation or peroxidation of unsaturated fatty acids in *P. microspore*, resulting in enhancement of membrane fluidity.

### Antifungal Effect of GO on Disease Development in Chinese Olive Fruits

**FIGURE 6:**
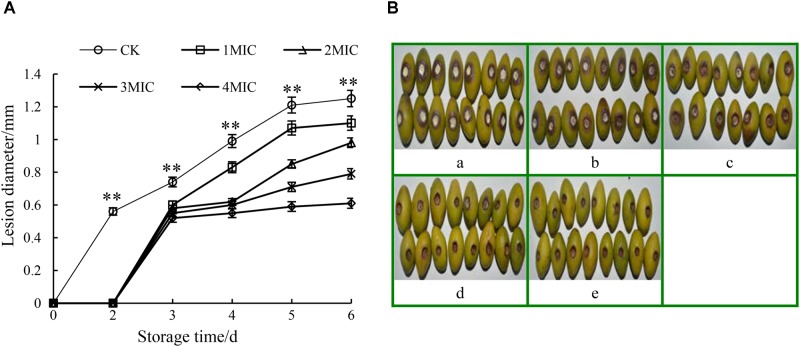
Effects of GO treatments on the lesion diameter **(A)** and lesion development **(B)** of *P. microspora*-infected Chinese olive fruits. Each value is the mean for three replicates. The vertical bar indicates the standard error. Photograph of a in Figure 6B represented the treatment of propylene glycol for control. Photographs of b to e represented the treatments of GO with concentration of 1 MIC, 2 MIC, 3 MIC, 4 MIC, respectively. The vertical bar indicates the standard error. The asterisks indicate significant difference between control and GO treated fruit (^∗∗^*P* < 0.01).

Compared to the control, the disease development of harvested Chinese olive fruit wounded-inoculated with *P. microspore* was remarkably suppressed (*p* < 0.01) by the treatments with GO (Figure 6). As shown in Figure 6A, the lesion diameter of *P. microspore*-infected Chinese olive fruits treated with GO was smaller than that of the control group (CK). The extension rate of lesion zone slowed down with the increase in the GO concentration, while no obvious increase was observed when the GO concentration exceeded 3 MIC. Meanwhile, the lesion zone of the fruits was accompanied by orange halo, pitted pericarp and covered with a large number of gray-white mycelia at the end of storage period, however, no mycelia growth and the lesion dried quickly on fruits when treated with concentration of 3 and 4 MIC GO (Figure 6B). Base on the above results, the optimal inhibitory concentration of GO for *P. microspore* infected Chinese olive fruits was 3 MIC.

## Conclusion

Ginger oleoresin effectively inhibited *in vitro* mycelial growth and spore germination of *P. microspora*, and exerted antifungal activity *via* membrane-targeted mechanism with alteration of membrane permeability, collapse of membrane integrity, and membrane lipid peroxidation. The GO treatments destroyed the morphology of the mycelia increasing the leakage of intercellular electrolytes, proteins, sugars, and nuclein of *P. microspore*, leading to lethal effects on the pathogen. Moreover, the GO treatments remarkably suppressed disease development in harvested Chinese olive fruit wounded-inoculated with *P. microspore*. In summary, GO could be a potentially effective alternative to the traditional fungicides against the postharvest pathogenic fungi of fruits and vegetables.

## Author Contributions

TC, SC, and HL conceived and designed the research. TC, BK, LD, and JL carried out the experiments and analyzed the data. TC, BK, and LZ wrote the manuscript. GC and ML edited the English language of the manuscript. HL and SC supervised the research. All authors discussed the results, provided critical feedback and contributed to the final manuscript.

## Conflict of Interest Statement

The authors declare that the research was conducted in the absence of any commercial or financial relationships that could be construed as a potential conflict of interest.

## References

[B1] Ashraf S. A. Al-Shammari E. Hussain T. Tajuddin S. Panda B. P. (2017). In-vitro antimicrobial activity and identification of bioactive components using GC-MS of commercially available essential oils in Saudi Arabia. *J. Food Sci. Technol.* 54 3948–3958. 10.1007/s13197-017-2859-2 29085137 PMC5643812

[B2] Bag A. Chattopadhyay R. R. (2015). Evaluation of synergistic antibacterial and antioxidant efficacy of essential oils of spices and herbs in combination. *PLoS One* 10:e0131321. 10.1371/journal.pone.0131321 26132146 PMC4488432

[B3] Bellik Y. (2014). Total antioxidant activity and antimicrobial potency of the essential oil and oleoresin of *Zingiber officinale* roscoe. *Asian Pac. J. Trop. Dis.* 4 40–44. 10.1016/S2222-1808(14)60311-X

[B4] Bradford M. M. (1976). A rapid and sensitive method for the quantitation of microgram quantities of protein utilizing the principle of protein-dye binding. *Anal. Biochem.* 72 248–254. 10.1016/0003-2697(76)90527-3 942051

[B5] Chaemsanit S. Matan N. Matan N. (2018). Effect of peppermint oil on the shelf-life of dragon fruit during storage. *Food Control* 90 172–179. 10.1016/j.foodcont.2018.03.001

[B6] Chang Q. Su M. H. Chen Q. X. Zeng B. Y. Li H. H. Wang W. (2017). Physicochemical properties and antioxidant capacity of Chinese Olive (*Canarium album* L.) cultivars. *J. Food Sci.* 82 1369–1377. 10.1111/1750-3841.13740 28494096

[B7] Chen N. Q. Chen Y. H. Lin H. T. Lin Y. F. Wang H. (2016a). Isolation and identification of the pathogen causing fruit rot in harvested Chinese olives. *Modern Food Sci. Technol.* 32 138–142. 10.13982/j.mfst.1673-9078.2016.10.022

[B8] Chen N. Q. Lin H. T. Chen Y. H. Lin Y. F. Wang H. (2016b). Biological characteristics of *Pestalotiopsis microspora*. *Storage Process* 16 5–10. 10.3969/j.issn.1009-6221.2016.03.002

[B9] Chen N. Q. Liu Y. M. Lin H. T. Kong X. J. Lin Y. F. Chen Y. H. (2015). Advances in post-harvest disease and storage technology of Chinese olive fruit. *Packag. Food Mach.* 33 49–53.

[B10] Hazzit M. Baaliouamer A. Faleiro M. L. Miguel M. G. (2006). Composition of the essential oils of thymus and origanum species from algeria and their antioxidant and antimicrobial activities. *J. Agric. Food Chem.* 54 6314–6321. 10.1021/jf0606104 16910725

[B11] He Z. Xia W. Liu Q. Chen J. (2008). Identification of a new phenolic compound from Chinese olive (*Canarium album* L.) fruit. *Eur. Food Res. Technol.* 228 339–343. 10.1007/s00217-008-0939-2

[B12] Jayasena D. D. Jo C. (2013). Potential application of essential oils as natural antioxidants in meat and meat products: a review. *Food Rev. Int.* 30 71–90. 10.1080/87559129.2013.853776

[B13] Kuo C. T. Liu T. H. Hsu T. H. Lin F. Y. Chen H. Y. (2015). Antioxidant and antiglycation properties of different solvent extracts from Chinese olive (*Canarium album* L.) fruit. *Asian Pac. J. Trop. Med.* 8 1013–1021. 10.1016/j.apjtm.2015.11.013 26706672

[B14] Li W. Yuan S. Sun J. Li Q. Jiang W. Cao J. (2018). Ethyl p-coumarate exerts antifungal activity in vitro and in vivo against fruit *Alternaria alternata* via membrane-targeted mechanism. *Int. J. Food Microbiol.* 278 26–35. 10.1016/j.ijfoodmicro.2018.04.024 29702314

[B15] Lin S. L. Chi W. W. Hu J. M. Pan Q. Zheng B. D. Zeng S. X. (2017). Sensory and nutritional properties of Chinese olive pomace based high fibre biscuit. *Emir. J. Food Agric.* 29 495–501. 10.9755/ejfa.2016-12-1908

[B16] Moshayedi S. Shahraz F. Schaffner D. W. Khanlarkhani A. Shojaee-Aliabadi S. Shahnia M. (2013). In vitro control of enterococcus faecalis by zataria multilfolira boiss, origanum vulgare l. and mentha pulegium essential oils. *J. Food Safety* 33 327–332. 10.1111/jfs.12056

[B17] Nanasombat S. Wimuttigosol P. (2011). Antimicrobial and antioxidant activity of spice essential oils. *Food Sci. Biotechnol.* 20 45–53. 10.1007/s10068-011-0007-8

[B18] Pane C. Fratianni F. Parisi M. Nazzaro F. Zaccardelli M. (2016). Control of alternaria post-harvest infections on cherry tomato fruits by wild pepper phenolic-rich extracts. *Crop Prot.* 84 81–87. 10.1016/j.cropro.2016.02.015

[B19] Shukla R. Kumar A. Singh P. Dubey N. K. (2009). Efficacy of Lippia alba (Mill.) N.E. *Int. J. Food Microbiol.* 135 165–170. 10.1016/j.ijfoodmicro.2009.08.002 19726096

[B20] Singh G. Kapoor I. P. Singh P. de Heluani C. S. de Lampasona M. P. Catalan C. A. (2008). Chemistry, antioxidant and antimicrobial investigations on essential oil and oleoresins of *Zingiber officinale*. *Food Chem. Toxicol.* 46 3295–3302. 10.1016/j.fct.2008.07.017 18706468

[B21] Tian J. Huang B. Luo X. L. Zeng H. Ban X. Q. He J. S. (2012). The control of *Aspergillus flavus* with cinnamomum jensenianum Hand-Mazz essential oil and its potential use as a food preservative. *Food Chem.* 130 520–527. 10.1016/j.foodchem.2011.07.061

[B22] Tian J. Wang Y. Z. Zeng H. Li Z. Y. Zhang P. Tessema A. (2015). Efficacy and possible mechanisms of perillaldehyde in control of *Aspergillus niger* causing grape decay. *Int. J. Food Microbiol.* 202 27–34. 10.1016/j.ijfoodmicro.2015.02.022 25755082

[B23] Varakumar S. Umesh K. V. Singhal R. S. (2017). Enhanced extraction of oleoresin from ginger (*Zingiber officinale*) rhizome powder using enzyme-assisted three phase partitioning. *Food Chem.* 216 27–36. 10.1016/j.foodchem.2016.07.180 27596388

[B24] Yamamoto-Ribeiro M. M. Grespan R. Kohiyama C. Y. Ferreira F. D. Mossini S. A. Silva E. L. (2013). Effect of *Zingiber officinale* essential oil on Fusarium verticillioides and fumonisin production. *Food Chem.* 141 3147–3152. 10.1016/j.foodchem.2013.05.144 23871071

[B25] Zhan M. M. Cheng Z. Z. Su G. C. Wang A. Y. Chen H. P. Shan Z. (2015). Genetic relationships analysis of olive cultivars grown in China. *Genet. Mol. Res.* 14 5958–5969. 10.4238/2015.June.1.13 26125795

[B26] Zhang C. Q. Liu Y. H. Wu H. M. Xu B. C. Sun P. L. Xu Z. H. (2012). Baseline sensitivity of *Pestalotiopsis microspora*, which causes black spot disease on Chinese hickory (*Carya cathayensis*), to pyraclostrobin. *Crop Prot.* 42 256–259. 10.1016/j.cropro.2012.07.018

